# The Relationship Between Food-Based Pro-inflammatory Diet and Sarcopenia: Findings From a Cross-Sectional Study in Iranian Elderly People

**DOI:** 10.3389/fmed.2021.649907

**Published:** 2021-05-10

**Authors:** Amir Bagheri, Rezvan Hashemi, Sanaz Soltani, Ramin Heshmat, Ahmadreza Dorosty Motlagh, Bagher Larijani, Ahmad Esmaillzadeh

**Affiliations:** ^1^Department of Community Nutrition, School of Nutritional Sciences and Dietetics, Tehran University of Medical Sciences, Tehran, Iran; ^2^Department of Geriatric Medicine, Ziaeian Hospital, Tehran University of Medical Sciences, Tehran, Iran; ^3^Chronic Diseases Research Center, Endocrinology and Metabolism Population Sciences Institute, Tehran University of Medical Sciences, Tehran, Iran; ^4^Endocrinology and Metabolism Research Center, Endocrinology and Metabolism Clinical Sciences Institute, Tehran University of Medical Sciences, Tehran, Iran; ^5^Obesity and Eating Habits Research Center, Endocrinology and Metabolism Molecular-Cellular Sciences Institute, Tehran University of Medical Sciences, Tehran, Iran; ^6^Department of Community Nutrition, Isfahan University of Medical Sciences, Isfahan, Iran

**Keywords:** food-based inflammatory potential of the diet, sarcopenia, muscle mass, muscle strength, gait speed

## Abstract

**Background:** Sarcopenia has rarely been linked to Food-based Inflammatory Potential of the Diet (FIPD) in earlier studies. This study was performed to examine the association of FIPD and sarcopenia and its components.

**Method:** In the cross-sectional research, dietary intakes of 300 randomly-selected elderly adults aged 55 years or older were collected through a validated food frequency questionnaire. We constructed FIPD score based on average consumptions of 28 food items. According to The European Working Group on Sarcopenia definition, sarcopenia and its components such as muscle strength, muscle mass, and gait speed were defined.

**Result:** No significant difference was found between the prevalence of sarcopenia (*P* = 0.05), low muscle mass (*P* = 0.27), low handgrip strength (*P* = 0.72), and lower gait speed (*P* = 0.14) across tertiles of FIPD score. Moreover, we did not find significant differences among means of handgrip strength (*P* = 0.65), muscle mass (*P* = 0.33), and walking speed (*P* = 0.89) across FIPD categories. However, binary logistic regression analysis indicated a significant positive relationship between FIPD score and odds of sarcopenia; such that subjects in the top vs. those in the bottom FIPD tertile had 155% greater chance of having sarcopenia (OR: 2.55; 95% CI: 1.17–5.55). After controlling for all confounding factors, this association strengthened (OR: 2.67; 95% CI: 1.18–6.01).

**Conclusion:** We found that greater FIPD score, which means a more pro-inflammatory diet, was positively linked with sarcopenia.

## Introduction

Sarcopenia is a generalized and progressive skeletal muscle disease that causes rapid loss of muscle health (1). It is related to chronic disease and known as a public health problem (1, 2). The projection for number of people with sarcopenia is its increasing rate from 50 million in 2010 to more than 200 million in 2050 worldwide (3). It is estimated that Iran's aging population will approximately reach 10% of the population in 2026 (4). Additionally, the prevalence of sarcopenia in Iran was 17 to 33% (5). Thus, diagnosis of underlying factor contributing to this condition is essential.

Aging, inactivity, poor diet, chronic disease, inflammation, and iatrogenic factors are the underlying causes of sarcopenia (1). Inflammation activates many molecular pathways involved in this disease by stimulating protein catabolism and suppressing muscle synthesis (6, 7). Therefore, high levels of inflammatory cytokines have a harmful effect on muscle strength and mass (8). Diet is a modifiable factor for both sarcopenia and inflammation (9). Although dietary inflammatory index (DII) has earlier been designed to evaluate the inflammatory properties of the diet, it mostly based on nutrients (10). There are the complex mixture of nutrients and chemicals in the diet and their effect on each other (11). This can distort the known potential of their anti- or pro-inflammatory nature. Thus, studies of single nutrients and even single foods may not fully address the complex nature of a diet (12). Therefore, the use of food-based inflammatory potential of the diet (FIPD) that considers foods together is suggested (13, 14). According to this idea, Tabung et al. constructed FIPD index based on dietary intakes of foods or food groups to survey the inflammatory capacity of the diet (14). This food-based dietary pattern could be conveniently interpreted for disease prevention and health improvement as dietary guidelines (14). Such dietary patterns have been shown to predict concentrations of plasma inflammatory factors better than DII (13).

Despite some studies on the link between food-based inflammatory potential of the diet (FIPD) and risk of some chronic disease including colorectal (15) and ovarian cancer (16), irritable bowel syndrome (17), and psychological illnesses (18), to our knowledge there is no study explored the association of this dietary index and risk of sarcopenia. Moreover, scarce data are available about the association of diet and sarcopenia in the Middle East. Therefore, this study was designed to examine the relationship between FIPD and sarcopenia and its components.

## Materials and Methods

### Participants

We carried out a population-based cross-sectional study from May to October 2011 in Tehran, Iran. Tehran is the largest city and capital of Iran. This metropolitan region accounts for 10.8% of Iran's total population (19). The data collection and sampling method with more details have earlier been published (20). Briefly, we recruited 300 elderly adults (150 women and 150 men) aged 55 years or older who were selected through a cluster random selection method in district 6 of Tehran. According to the postal code address, the head of each cluster was determined. It should be noted that age and gender distribution, as well as demographic status in the population of district 6 of Tehran, where the current study's population came from, are representative of Tehran's population (19). To avoid heterogeneity in the data, persons whose major cause of sarcopenia did not due to aging were not included. Subjects who had sarcopenia due to lack of moving as well as those with artificial limbs or limb prostheses were not included. In addition, subjects with debilitating diseases (for instance malignancy and organ failure) that might predispose an individual to sarcopenia were not included as well. Tehran University of Medical Sciences Ethics' Committee accepted the protocol study. First, the settings of study were elucidated to the participants and then the informed written consent were obtained.

### Dietary Intake Assessment

Dietary data of study participants were gathered with a Block-format 117-item Food Frequency Questionnaire (FFQ), for which detailed information about the validity and reliability has been given elsewhere (20, 21). Trained nutritionist administered the FFQs through in-person meeting. The questionnaire contained a list of food items that their portion sizes were specified. The booklet of “household measures” was used to convert the dietary items to grams per day. We calculated daily nutrients and energy intake of each participant with Iranian modified version of Nutritionist IV software.

### Development of a Food-Based Inflammatory Potential of Diet

For construction of FIPD, we used the earlier database on 486 Tehrani female adults about the association of food and food groups contributed to systemic inflammation (22). In the mentioned study, the level of inflammatory markers was assessed with serum interleukin-6 (IL-6), Tumor Necrosis Factor alpha (TNF alpha), and high-sensitivity C-reactive protein (hs-CRP) level (22). Dietary items that loaded in the western dietary pattern were determined as pro-inflammatory foods and other food items loaded in the healthy dietary pattern were determined as anti-inflammatory items. We used this approach because that study observed the healthy dietary pattern could significantly decrease inflammatory markers as well as the western dietary pattern could significantly elevated inflammatory markers (22). First, average daily consumptions of 28 food groups including 12 anti-inflammatories (e.g., poultry, fish, tomatoes, legumes, yellow vegetables, cruciferous vegetables, other vegetables, fruits, fruit juices, green leafy vegetables, tea, and whole grains) and 16 pro-inflammatory items (eggs, dairy, potatoes, pizza, butter, red meats, coffee, French fries, sweets and desserts, refined grains, snacks, hydrogenated oils, processed meats, hydrogenated fats, soft drinks, and mayonnaise) were calculated. Then, amounts of these foods items were controlled for energy with the residual analysis (23). For each individual, we multiplied average daily consumption of each food items through the factor loadings attained in the aforementioned paper (22). Then, total FIPD score of each individual was calculated through summation of each dietary item scores. Lastly, to decrease the magnitude of the scores, the final FIPD score was divided by 100. A lower FIPD or more negative score shows a less pro-inflammatory diet, and vice versa.

### Assessment of Sarcopenia

Sarcopenia was determined according to the definition suggested by the European Working Group on Sarcopenia (EWGSOP) (3). We considered the combination of both low muscle mass and either weak grip strength or slow gait speed to determine sarcopenia. The ratio of whole lean mass of arms and legs of each individuals known as ASM (Appendicular Skeletal Muscle) (24) divided to their height^2^ (ASM/height^2^) was considered as the muscle mass. A dual-energy X-ray absorptiometry (DEXA) (Discovery W S/N 84430) was applied for this assessment. The muscle mass lower than 5.45 kg/m^2^ for women and lower than 7.26 kg/m^2^ for men were diagnosed as low muscle mass (3).

Muscle strength of each participant was determined by handgrip test. A pneumatic squeeze bulb dynamometer (named: c7489-02 Rolyan) adjusted in pound per inch^2^ (psi) was used to obtained handgrip strength. Participants had to squeeze 3 times for both hands with a 30 s rest after each squeeze. Next, the mean result of all measurement was recorded. Finally, the handgrip strength lower than 30 kg for men and lower than 20 kg for women was determined as low muscle strength (25). We applied a 4-m walk gait speed exam to evaluate physical performance of participants (3). Participants with gait speeds < 0.8 m/s was diagnosed as slow gait speed (3).

### Assessment of Other Variables

Required information about non-dietary data including age, sex, socio-economic status, alcohol consumption, and smoking habits were obtained with a general questionnaire. The former medical history including history of arthritis, stroke, asthma, myocardial infarction, and diabetes, and also a history of medicine use including statins, insulin angiotensin-converting enzyme inhibitors, sexual hormones, and corticosteroid were collected as well. The physical activity performance was evaluated by the short form of the International Physical Activity Questionnaire (IPAQ) (26). Next, in accordance with recommendation of IPAQ, we calculated a metabolic equivalent-hour per week (MET-h/week) for all contributors (27). We used a digital scale to measure weight while participants were minimally clothed. We measured height in a standing position without shoes by a wall tape meter. While contributors were standing and usually breathe, their waist circumference was measured in the middle of the iliac crest and lower rib margin. Weight (kg) divided by height squared (m2) was applied to compute body mass index (BMI).

### Statistical Analysis

Participants were categorized based on tertile cut-off points of FIPD score. We used tertiles instead of quartiles or quintiles due to not having a large sample size. In addition, we reached the best associations when we considered participants across tertiles of FIPD score. To examine the differences in distribution of the participant's characteristics across tertiles of FIPD, we used Chi-square and ANOVA analyses for categorical and continuous variables, respectively. Dietary intakes were assessed through ANCOVA across FIPD score categories, in which we adjusted for sex, age, and energy. Binary logistic regression was applied to find the link of FIPD score with sarcopenia in crude and multivariable-adjusted models. In the first model, energy intake (kcal/d), sex (male/female), and age (continuous) were controlled for. In the second model, further adjustments were performed for alcohol consumption (yes/no), smoking (yes/no), history of chronic illness (cerebrovascular accident, arthritis, asthma, diabetes, and myocardial infarction), physical activity (MET-h/wk), and medication use (corticosteroid, statin, testosterone, and estrogen). Moreover, the odds ratio for sarcopenia in the second and third tertiles were compared with the lowest tertile (reference category). We included the tertile categories of the FIPD score as an ordinal variable in the logistic regression analysis to attain *P* for trends. All aforementioned analyses were done with SPSS (version 26). *P*-values lower than 0.05 were considered significant.

## Results

In the present investigation, total FIPD score was between −48.4 and −0.45. The cut-off points of FIPD score across increasing tertiles was <-11.25, −11.25 to −7.37, and >-7.37, respectively. Distribution of participants as well as mean values of continuous variables across tertiles of FIPD is shown in Table 1. Subjects in the third tertile compared to subjects in the first tertile of FIPD had less physical activity (*P* = 0.005). No significant difference was detected in mean age and BMI across FIPD categories. The distribution of subjects in alcohol use, smoking, medical or medication history between the highest vs. lowest categories of FIPD was not significantly different.

**Table 1:** Characteristics of study participants in FIPD categories^*^.

	**Tertiles of FIPD score**			***P*^**†**^**
	**T_**1**_ (*n* = 100)**	**T_**2**_ (*n* = 100)**	**T_**3**_ (*n* = 100)**	
FIPD range	<-11.25	−11.25, −7.37	>-7.37	
Age (y)	66.33 ± 7.50	66.82 ± 7.37	67.24 ± 8.26	0.70
BMI (kg/m^2^)	27.51 ± 4.12	27.28 ± 4.47	27.34 ± 4.05	0.92
Physical activity (MET-h/w)	1626.1 ± 1665.9	1279.71 ± 1358.2	977.7 ± 1157.3	0.005
Female (%)	55	49	49	0.61
Alcohol use (%)	12	16	12	0.63
Smoking (%)	13	15	10	0.56
**Medical history**				
Yes (%)	16	20	16	0.68
No (%)	84	80	84	
**Drug history**				
Sexual hormone use (%)	2	3	4	0.70
Statin use (%)	41	30	39	0.22
Corticosteroid use (%)	3	1	4	0.40

* *All values are mean ± SD, unless indicated*.

† *P-value for quantitative variables and qualitative variables were obtained from ANOVA and chi-square, respectively*.

*FIPD, food-based inflammatory potential of diet*.

Age-, gender- and energy-adjusted means of selected food items throughout FIPD categories are displayed in Table 2. We observed a higher score of FIPD significantly related with a lower intake of cruciferous vegetables, fruits, yellow vegetables, green leafy vegetables, tomatoes, other vegetables, fish, tea, and low-fat dairy. Moreover, subjects in the highest rank of FIPD score had significantly greater consumptions of refined grains, high-fat dairy, soft drinks, red meats, potatoes, and French fries than subjects in the lowest rank.

**Table 2:** Dietary intakes of study participants by FIPD categories^*^.

**Variables**	**Tertiles of FIPD score**			***P*^**†**^**
	**T_**1**_ (*n* = 100)**	**T_**2**_ (*n* = 100)**	**T_**3**_ (*n* = 100)**	
Fruits (g/d)	861.41 ± 21.09	589.92 ± 20.58	465.11 ± 20.74	< 0.001
Fruit juices (g/d)	52.53 ± 7.63	36.12 ± 7.45	35.72 ± 7.51	0.21
Poultry (g/d)	26.30 ± 1.80	23.33 ± 1.75	22.61 ± 1.77	0.32
Legumes (g/d)	37.10 ± 3.37	45.93 ± 3.29	39.17 ± 3.31	0.14
Cruciferous vegetables (g/d)	14.57 ± 1.71	9.65 ± 1.67	7.90 ± 1.68	0.02
Green leafy vegetables (g/d)	44.30 ± 2.64	30.06 ± 2.58	27.66 ± 2.60	< 0.001
Yellow vegetables (g/d)	35.68 ± 2.23	23.75 ± 2.18	19.53 ± 2.20	< 0.001
Other vegetables (g/d)	454.95 ± 14.75	296.19 ± 14.39	241.03 ± 14.51	< 0.001
Tea (g/d)	910.52 ± 56.28	839.30 ± 54.92	578.18 ± 55.36	< 0.001
Tomatoes (g/d)	220.91 ± 9.78	153.80 ± 9.54	115.61 ± 9.62	< 0.001
Whole grains (g/d)	111.47 ± 8.60	93.62 ± 8.39	98.20 ± 8.46	0.32
Butter (g/d)	2.89 ± 0.45	1.79 ± 0.43	2.61 ± 0.44	0.19
Potatoes (g/d)	18.48 ± 3.26	31.20 ± 3.18	31.55 ± 3.20	0.007
Low fat dairy (g/d)	314.51 ± 22.81	254.65 ± 22.25	192.60 ± 22.43	0.001
High fat dairy (g/d)	240.39 ± 19.96	313.28 ± 19.48	362.44 ± 19.63	< 0.001
Fish (g/d)	20.26 ± 2.36	12.27 ± 2.30	11.67 ± 2.32	0.01
Refined grains (g/d)	137.03 ± 14.30	195.69 ± 13.95	287.01 ± 14.07	< 0.001
Red meats (g/d)	29.12 ± 2.96	34.70 ± 2.88	42.41 ± 2.91	0.007
Processed meats (g/d)	1.11 ± 0.73	2.58 ± 0.71	3.01 ± 0.71	0.16
Sweets and desserts (g/d)	6.16 ± 0.96	6.15 ± 0.94	6.96 ± 0.95	0.79
Pizza (g/d)	7.97 ± 1.75	4.81 ± 1.71	6.60 ± 1.73	0.44
Eggs (g/d)	18.99 ± 2.82	15.48 ± 2.76	14.71 ± 2.78	0.53
Soft drinks (g/d)	22.27 ± 7.66	25.80 ± 7.47	54.81 ± 7.53	0.004
French fries (g/d)	2.76 ± 0.54	3.40 ± 0.53	4.65 ± 0.53	0.04
Coffee (g/d)	31.51 ± 6.39	19.59 ± 6.23	13.30 ± 6.28	0.13
Mayonnaise (g/d)	1.80 ± 0.49	1.93 ± 0.48	2.47 ± 0.48	0.59
Hydrogenated fats (g/d)	2.10 ± 0.97	5.04 ± 0.94	3.80 ± 0.95	0.10
Vegetables oils (g/d)	7.51 ± 0.59	6.81 ± 0.58	6.87 ± 0.58	0.66

* *All values are mean ± SE; all values are adjusted for energy intake, sex, and age*.

† *ANCOVA for all variables*.

*FIPD, food-based inflammatory potential of diet*.

Table 3 indicates the distribution of sarcopenia, low muscle mass, low muscle strength, and low gait speed across FIPD categories. We did not find a significant difference in the distribution of sarcopenia (*P* = 0.05), low muscle mass (*P* = 0.27), low handgrip strength (*P* = 0.72), and lower gait speed (*P* = 0.14) throughout categories of FIPD score. In addition, no significant differences were found among means of handgrip strength, muscle mass, and walking speed across FIPD categories even after adjusting for all covariates.

**Table 3:** Distribution of sarcopenia and its components across tertile FIPD categories.

	**Tertiles of FIPD score**			P^*^
	**T_**1**_ (*n* = 100)**	**T_**2**_ (*n* = 100)**	**T_**3**_ (*n* = 100)**	
Sarcopenia (%)	11	19	24	0.05
Low muscle mass (%)^†^	34	38	45	0.27
Low hand grip strength (%)^‡^	35	30	31	0.72
Low gait speed (%)^§^	33	43	46	0.14
Means of components				
**Muscle mass [ASM/h2] (kg)**				
Crude	6.62 ± 0.09	6.67 ± 0.09	6.53 ± 0.09	0.63
Model 1	6.66 ± 0.08	6.64 ± 0.08	6.51 ± 0.08	0.39
Model 2	6.67 ± 0.08	6.65 ± 0.08	6.51 ± 0.08	0.33
**Hand grip strength (psi)**				
Crude	10.77 ± 0.35	11.38 ± 0.35	10.98 ± 0.35	0.47
Model 1	10.92 ± 0.24	11.28 ± 0.23	10.93 ± 0.23	0.48
Model 2	10.91 ± 0.24	11.22 ± 0.23	11.02 ± 0.24	0.65
**Gait speed (m/s)**				
Crude	0.85 ± 0.02	0.84 ± 0.02	0.83 ± 0.02	0.71
Model 1	0.85 ± 0.02	0.83 ± 0.02	0.83 ± 0.02	0.74
Model 2	0.85 ± 0.02	0.84 ± 0.02	0.85 ± 0.2	0.89

* *P-value for quantitative variables and qualitative variables were obtained from ANCOVA and chi-square, respectively (P < 0.05 significant)*.

† *Muscle mass < 5.5 (kg/m^2^) for women and < 7.0 (kg/m^2^) for men (3)*.

‡ *Muscle strength < 30 kg for men and < 20 kg for women (25)*.

§ *Gait speeds ≤ 0.8 m/s (3)*.

*Model 1: Adjusted for energy, age and sex*.

*Model 2: Further adjusted for smoking, physical activity, medication use (estrogen, testosterone, corticosteroid, and statin), alcohol consumption, and history of disease*.

*FIPD, food-based inflammatory potential of diet*.

Odds ratios (ORs) and 95% CIs from crude and multivariable-adjusted model for sarcopenia across FIPD tertiles are depicted in Table 4. A significant relationship between a higher FIPD score and increasing odds of sarcopenia (OR: 2.55; 95% CI: 1.17–5.55) was identified in the unadjusted model. After controlling for energy intake, sex, and age (model 1), the relationship remained significant (OR: 2.67; 95% CI: 1.18–6.01). Even after additional controlling for all covariates, we found that subjects in the top vs. the bottom FIPD tertile had higher odds of sarcopenia (OR: 2.57; 95% CI: 1.11–5.89).

**Table 4:** Multivariable-adjusted odds ratios (95% CIs) for sarcopenia across tertile FIPD categories.

	**Tertiles of FIPD score**			***P* trend**
	**T_**1**_**	**T_**2**_**	**T_**3**_**	
Sarcopenia				
*n* (case)	11	19	24	
Crude	1	1.89 (0.85–4.22)	2.55 (1.17–5.55)	0.01
Model 1	1	1.97 (0.86–4.51)	2.67 (1.18–6.01)	0.01
Model 2	1	2.06 (0.88–4.82)	2.57 (1.11–5.89)	0.02

*Model 1: Adjusted for energy, age and sex. Model 2: Further adjusted for smoking, physical activity, medication use (estrogen, testosterone, corticosteroid, and statin), alcohol consumption, and history of disease*.

*FIPD, food-based inflammatory potential of diet*.

## Discussion

We evaluated the relationship between the food-based inflammatory potential of diet and sarcopenia and its components. A higher FIPD score or a more pro-inflammatory diet was linked with elevated likelihood of sarcopenia. Nevertheless, we did not find any relationship between FIPD and low gait speed, low muscle strength, and low muscle mass. As far as we know, this is the first study elucidate the role of the food-based inflammatory score in sarcopenia.

Sarcopenia is known as a disease, associated with increment hazard of disability, mortality, and fall (28, 29). It appears that level of inflammatory marker has been increased during the aging period that might cause muscle weakness or sarcopenia (30); however, dietary intakes can also contribute to elevated inflammation and muscle deterioration (9). FIPD was validated and expanded to clarify the whole dietary inflammatory capacity (14). We found that subjects with high FIPD score had a greater chance of having sarcopenia. It must be mentioned that data are very limited on the association between diet and sarcopenia especially about the inflammatory capacity of the diet. A large cross-sectional study from the United State demonstrated that higher nutrient-based DII score was related to increased risk of sarcopenia (31). Our recent publication in the current population revealed that nutrient-based DII was positively associated with sarcopenia (32). However, the current analysis is different from previous ones because unlike previous one (32), here we considered foods to compute dietary inflammatory capacity. Although both DII based on nutrient and food-based FIPD indexes evaluate the inflammatory properties of diet, nutrient-based index might have some limitations to assess this potential due to the complex synergistic and interactions effect of nutrients and chemicals in the diet (11, 14). The FIPD is a novel index constructed based on foods and food groups and is more appropriate for clinical practice and public recommendations (14). Earlier studies have shown that the food-based assessment of dietary inflammatory potential predicts systemic inflammation better than nutrient-based DII (13). However, additional investigation is needed to elucidate the association between dietary inflammatory capacity and components of sarcopenia.

As expected, we found individuals with high adherence to pro-inflammatory diet (the top FIPD tertile) had significantly lower consumptions of food groups with anti-inflammatory properties including fish, tomatoes, cruciferous vegetables, yellow vegetables, green leafy vegetables, other vegetables, fruits, tea, and low-fat dairy. Additionally, subjects in the third vs. the first FIPD tertile had significantly greater consumptions of potatoes, high-fat dairy, refined grains, red meats, soft drinks, and French fries. Based on these findings, a dietary pattern with a high intake of fish, vegetables, fruit, and low-fat dairy along with a fewer intake of red meats, refined grains, potatoes, high-fat dairy, and soft drinks might be beneficial for maintaining muscles. In line with this results, a cohort study conducted in England, Wales, and Scotland reported that a healthy dietary pattern (means greater consumption of leafy vegetables, fresh fruit, and whole-grain bread, but lesser consumption of processed meat, white bread, and added sugar) in adulthood (ages 36, 43, 53) was linked with improved muscle performance in older age (60–64) (33). A Swedish cohort study (34), that investigated the relationship of dietary patterns at study baseline with average age 71 years, suggested that eating a healthy dietary pattern especially “the Mediterranean diet” tended to reduce the progress of sarcopenia 16 years later.

There is growing evidence that increasing pro-inflammatory markers such as TNF-α, IL-1β, IL-6 are the main cause of skeletal muscle wasting and sarcopenia (35). High serum levels of these inflammatory markers can negatively influence skeletal muscle by inhibiting the expression and activity of GH and IGF-I (35, 36). Of note, studies conducted to determine the effect of anti-inflammatory medications on skeletal muscle and inflammation, showed that these anti-inflammatory drugs can significantly reduce loss of muscle mass and keep muscle strength (37). It appears that our findings provide further evidence on the contribution of dietary inflammatory potential to sarcopenia.

This study had some strengths and limitations. For the first time, we used a novel food-based IPD to predict sarcopenia and its components. Evidence found that FIPD better predicts the body's inflammation and its related co-morbidities than nutrient-based dietary inflammatory index (13). Several potential confounders were considered in the analyses. Moreover, we used a validated FFQ for the evaluation of participants dietary intakes. Along with strengths, our study had some limitations as well. First, the cross-sectional design of the study prohibits us to reach a causal relationship, because longitudinal data is not available as well as exposure and outcome are identified in each individual simultaneously. Second, the possibility of misclassification of study subjects, due to measurement errors, cannot be avoided. Third, despite considering numerous confounding factors, residual confounders cannot be ignored. Fourth, we did not measure inflammatory cytokines in the current study. The FIPD was constructed based on factor loadings of food items in healthy or Western dietary patterns among Iranian female teachers, not the current study population. It is better to construct FIPD based on coefficients between food groups and inflammatory biomarkers obtained in the same study population. Finally, the study was performed on a small sample (maximum 300 cases), due to inaccessibility to more DEXA machine in Tehran and budget limitations. Thus, caution is required to the extrapolation of our findings to the total Iranian population.

## Conclusion

Findings from this population-based cross-sectional investigation support the role of food-based inflammatory potential of the diet in sarcopenia. Strategies to reduce consumption of foods with a greater pro-inflammatory capacity along with increasing consumption of foods with anti-inflammatory features may have benefits for older adults to prevent muscle loss. Prospective studies however are needed to verify these findings.

## Data Availability Statement

The raw data supporting the conclusions of this article will be made available by the authors, on reasonable request to the corresponding author.

## Ethics Statement

The studies involving human participants were reviewed and approved by Tehran University of Medical Sciences Ethics' Committee. The patients/participants provided their written informed consent to participate in this study.

## Author Contributions

AB, RHa, AD, BL, RHe, SS, and AE participated to the data collection, statistical analyses, design, conception, data explanation, manuscript writing, and approval of the final version of the manuscript. All authors contributed to the article and approved the submitted version.

## Conflict of Interest

The authors declare that the research was conducted in the absence of any commercial or financial relationships that could be construed as a potential conflict of interest.
